# Prognostic Factors in Anti-glomerular Basement Membrane Disease: A Multicenter Study of 119 Patients

**DOI:** 10.3389/fimmu.2019.01665

**Published:** 2019-07-18

**Authors:** Cindy Marques, Julien Carvelli, Lucie Biard, Stanislas Faguer, François Provôt, Marie Matignon, Jean-Jacques Boffa, Emmanuelle Plaisier, Alexandre Hertig, Maxime Touzot, Olivier Moranne, Xavier Belenfant, Djillali Annane, Thomas Quéméneur, Jacques Cadranel, Hassan Izzedine, Nicolas Bréchot, Patrice Cacoub, Alexis Piedrafita, Noémie Jourde-Chiche, David Saadoun

**Affiliations:** ^1^Inflammation-Immunopathology-Biotherapy Department (DHU i2B), Sorbonne Université, UPMC Univ Paris 06, UMR 7211, Paris, France; ^2^INSERM, UMR_S 959, Paris, France; ^3^CNRS, FRE3632, Paris, France; ^4^AP-HP, Groupe Hospitalier Pitié-Salpêtrière, Department of Internal Medicine and Clinical Immunology, Paris, France; ^5^Centre de Référence des Maladies Auto-Immunes et Systémiques Rares, Centre de Référence des Maladies Auto-Inflammatoires et de l'Amylose, Paris, France; ^6^Aix-Marseille Univ, APHM, C2VN, INRA 1260, INSERM 1263, CHU de la Conception, Centre de Néphrologie et Transplantation Rénale, Marseille, France; ^7^Department of Biostatistics and Medical Information, INSERM UMR1153 ECSTRRA Team, Hôpital Saint Louis, AP-HP, Paris, France; ^8^Département de Néphrologie et Transplantation d'organes, Centre de référence des maladies rénales rares, Hôpital Rangueil, CHU de Toulouse, Toulouse, France; ^9^Department of Nephrology, Centre Hospitalier Régional Universitaire de Lille, Lille, France; ^10^Department of Nephrology and Renal Transplantation, Groupe Hospitalier Henri-Mondor, AP-HP, Créteil, France; ^11^Sorbonne Université, UPMC Université Paris 06, Hôpital Tenon, Urgences Néphrologiques et Transplantation Rénale, Paris, France; ^12^AURA Paris Plaisance, Paris, France; ^13^Service Néphrologie-Dialyses-Aphérèse, Hôpital Caremeau, CHU Nîmes, et Faculté de Médecine Université de Montpellier-nimes, Nîmes, France; ^14^Nephrology and Dialysis, Centre Hospitalier Intercommunal André Grégoire, Montreuil, France; ^15^General ICU, Hôpital Raymond Poincaré, AP-HP, Garches, France; ^16^Department of Internal Medicine, Centre Hospitalier, Valenciennes, France; ^17^Chest Department and Constitutive Center for Rare Pulmonary Disease, Hôpital Tenon, AP-HP; Inflammation-Immunopathology-Biotherapy Department (DHU i2B) and Sorbonne Université, Paris, France; ^18^Department of Nephrology, Peupliers Private Hospital, Ramsay Générale de Santé, Paris, France; ^19^Medical-Surgical Intensive Care Unit, AP-HP, Groupe Hospitalier Pitié-Salpêtrière, Paris, France

**Keywords:** anti-glomerular basement membrane disease, Goodpasture's disease, glomerulonephritis, vasculitis, outcome, mortality

## Abstract

We report the overall and renal outcome in a French nationwide multicenter cohort of 119 patients with anti-glomerular basement membrane (anti-GBM) disease. Sixty-four patients (54%) had an exclusive renal involvement, 7 (6%) an isolated alveolar hemorrhage and 48 (40%) a combined renal and pulmonary involvement. Initial renal replacement therapy (RRT) was required in 78% of patients; 82% received plasmapheresis, 82% cyclophosphamide, and 9% rituximab. ANCA positive (28%) patients were older (70 vs. 47 years, *p* < 0.0001), less frequently smokers (26 vs. 54%, *p* = 0.03), and had less pulmonary involvement than ANCA- patients. The 5 years overall survival was 92%. Risk factors of death (*n* = 11, 9.2%) were age at onset [HR 4.10 per decade (1.89–8.88) *p* = 0.003], hypertension [HR 19.9 (2.52–157 0.2) *p* = 0.005], dyslipidemia [HR 11.1 (2.72–45) *p* = 0.0008], and need for mechanical ventilation [HR 5.20 (1.02–26.4) *p* = 0.047]. The use of plasmapheresis was associated with better survival [HR 0.29 (0.08–0.98) *p* = 0.046]. At 3 months, 55 (46%) patients had end-stage renal disease (ESRD) vs. 37 (31%) ESRD-free and 27 (23%) unevaluable with follow-up < 3 months. ESRD patients were older, more frequently female and had a higher serum creatinine level at presentation than those without ESRD. ESRD-free survival was evaluated in patients alive without ESRD at 3 months (*n* = 37) using a landmark approach. In conclusion, this large French nationwide study identifies prognosis factors of renal and overall survival in anti-GBM patients.

## Introduction

Anti-glomerular basement membrane (anti-GBM) disease is a rare small vessel vasculitis that affects the capillary beds of the kidneys and lungs (1). It is an organ-specific autoimmune disease mediated by circulating autoantibodies directed against the non-collagenous domain of the α3 chain of type IV collagen [α3(IV)NC1] (2–5). Clinical presentation, related to the involvement of both glomerular and alveolar membranes, includes rapidly progressive glomerulonephritis and pulmonary hemorrhage. A majority of patients with anti-GBM have both pulmonary and renal involvement, but 20–40% and <10% of patients have kidney or pulmonary involvement only, respectively. Twenty-one to 47% of patients also have antineutrophil cytoplasmic antibodies (ANCA) (6–10). They mostly display anti-myeloperoxidase (MPO) specificity (11, 12) and could be older than those with anti-GBM positivity alone (13), with a male preponderance (9).

The standard treatment for anti-GBM relies on plasma exchanges to rapidly remove pathogenic autoantibodies, combined with glucocorticoids and cyclophosphamide (CYC) (14). CYC is most often administered orally but some protocols include intravenous administration. Despite the lack of randomized controlled studies given the rarity and severity of the disease, the use of combination therapy has been the gold standard since the 1970s. According to the severity of the clinical course, some patients will require prolonged treatment with immunosuppressive drugs for as long as 6–12 months. Moreover, the addition of anti-CD20 rituximab monoclonal antibody therapy (375 mg/m^2^/week for 4 weeks) has been proposed for patients with severe and/or refractory anti-GBM disease (15). Similarly, the use of mycophenolate mofetil and cyclosporine has been reported in individual cases or small series (16–18).

Given the small number of large and homogeneous cohorts, few data are available on prognostic factors for renal and overall long-term evolution. A large Chinese study of 221 patients confirmed that the combination of plasmapheresis and corticosteroids correlated with overall and renal survival (19). A British study from 2015 showed that short-term renal survival was determined by the severity of initial renal impairment (oliguria and percentage of histological crescents); and that age, ANCA positivity, oliguria, and the presence of comorbidities were predictive of overall survival (OS) (13). In a recent study from the French Society of Hemapheresis, renal survival was only predicted by the severity of the renal presentation (20).

The present study was undertaken to report the outcome of anti-GBM. We compared anti-GBM patients according to ANCA status, and analyzed prognostic factors of overall and renal survival in a French nationwide cohort of 119 patients with anti-GBM disease.

## Methods

### Patients

We retrospectively reviewed the data of patients with anti-GBM disease diagnosed in 16 French centers between 1981 and 2017. Diagnosis of anti-GBM was based on the presence of circulating anti-GBM antibodies detected by ELISA or immunofluorescence and/or linear IgG fluorescence along the GBM on renal biopsy, which is the gold-standard for diagnosis of anti-GBM disease (21). A diagnosis of pulmonary hemorrhage was retained in patients with overt hemoptysis and/or pulmonary interstitial opacities on chest computed tomography (CT) and/or proven alveolar hemorrhage on bronchoalveolar lavage. Relapses were defined as pulmonary (i.e., recurrence of hemoptysis) and/or renal worsening (i.e., increase in serum creatinine level and proteinuria) more than 3 months after diagnosis elevation of anti-GBM autoantibodies and/or compatible renal biopsy. Before 3 months, we considered that it was a worsening of the disease. Included patients did not belong to the cohort-based study from the French Society of Hemapheresis (20). The study was approved by the ethical committee of Pitié-Salpêtrière University Hospital.

### Data Collection

Demographic data, medical history, clinical, biological, radiological, and histological data at presentation were collected. Intensive care stays, number of plasma exchanges as well as number and dose of different treatments regimen, were also reported. End-stage renal disease (ESRD) was defined as the persistence of renal failure with anuria or estimated glomerular filtration rate <15 ml/min/1.73 m^2^ after 3 months of evolution. Finally, overall and renal survival data up to 60 months of follow-up, adverse events, kidney or pulmonary transplants and relapses were also collected.

### Statistical Analyses

For description according to ANCA status and to renal status at M3, quantitative variables were compared with the Wilcoxon test or Kruskal-Wallis test when appropriate. Qualitative variables were compared with the Fisher test or the χ^2^ test when appropriate. Overall survival (OS) was defined as the time from the date of diagnosis to the date of death or last follow-up. Renal survival (RS) was examined both in the global population at M3 (3 months after the initial hospitalization), as a categorical endpoint, and in patients without ESRD alive at M3, as a time-to-event endpoint (ESRD-free survival) using a landmark approach (22). ESRD-free survival was defined as the time from M3 (confirmation if ESRD- profile) to the date of first ESRD diagnosis, death or last follow-up, whichever occurred first. Time-to-event outcomes were estimated using the Kaplan-Meier method. Univariate analyses of factors associated with survival outcomes were performed in Cox regression models, or using the LogRank test when appropriate. The proportional hazards assumption and loglinearity assumption for quantitative variables were assessed.

Tests were two-sided and a significance level smaller than 0.05 was considered to indicate a significant association. Analyzes were carried out with the statistical software R, version 3.4.1 (https://cran.r-project.org/).

## Results

### Characteristics of Anti-GBM Patients

The main clinical, laboratory, pathological, and immunological features are summarized in Table 1. We included 119 patients with a male to female ratio of 1 (60:59). The median age at the time of diagnostic was 54 years (range: 5–86) following a bimodal distribution with a first peak during the third decade and a second one around the age of 60. Fifty patients (42%) patients were smokers. Twelve patients (10%) reported a toxic exposure in the weeks preceding the onset of symptoms such as cannabis, ecstasy, pesticides, or cleaner household product. Twelve patients (10%) had a personal history of autoimmune or inflammatory disease including systemic scleroderma or Hashimoto thyroiditis; or vasculitis such as Takayasu arteritis.

**Table 1:** Characteristics of 119 anti-GBM patients at presentation.

**Clinical features**	
Age (years, median [IQR])	54 [29; 72]
Female (%)	59 (50)
Ethnic group^*^	
Caucasian (%)	94 (83)
Other (%)	19 (17)
Toxics	
Tobacco (%)^*^	50 (46)
Cannabis (%)^*^	6 (6)
Other (%)	12 (10)
Comorbidities	
Hypertension (%)^*^	40 (34)
Diabetes (%)^*^	9 (8)
Dyslipidemia (%)^*^	14 (12)
Time between onset and diagnosis (months, median [IQR])	0.4 [0.1; 0.9]
Symptom leading to the medical consultation^*^	
Fatigue (%)	38 (33)
Fever (%)	10 (9)
Dyspnea (%)	11 (10)
Cough (%)	7 (6)
Hemoptysis (%)	15 (13)
Microscopic hematuria (%)	9 (8)
Biological anomaly (%)	25 (22)
**Biological features**	
ANCA positivity (%)^*^	30 (28)
Hemoglobin level (g/dl, median [IQR])^*^	9 [8; 10]
CRP (mg/L, median [IQR])^*^	93 [38; 164]
**Renal involvement**	
Acute renal failure (%)^*^	101 (91)
Serum creatinine (mg/dl, median [IQR])^*^	7.2 [4.2; 11.4]
Proteinuria (> 0.5 g/dl, %)^*^	72 (91)
Microscopic hematuria (%)^*^	81 (98)
Leukocyturia (%)^*^	42 (93)
Serum albumin (g/l, median [IQR])^*^	27 [22; 31]
Renal biopsy (%)^*^	101 (86)
Extracapillary proliferation (%)^*^	69 (68)
Capsular rupture (%)^*^	32 (76)
Interstitial fibrosis (%)^*^	38 (64)
Hyaline thrombi (%)^*^	11 (15)
Immunofluorescence positivity (%)^*^	91 (99)
**Pulmonary involvement**	
Dyspnea (%)^*^	42 (38)
Cough (%)^*^	39 (35)
Overt hemoptysis (%)^*^	31 (27)
Pulmonary interstitial opacities on chest CT (*n*, %)^*^	40 (57)
Alveolar hemorrhage on bronchoalveolar lavage (*n*, %)^*^	23 (92)
PaO2 (mmHg, median [IQR])^*^	77 [60; 86]
**Therapeutic regimens**	
Admission to intensive care (%)^*^	36 (31)
Mechanical ventilation (%)	8 (22)
Initial hemodialysis (%)^*^	91 (78)
Plasmapheresis (%)^*^	97 (82)
Corticosteroid pulses (%)^*^	81 (70)
Oral corticosteroids (%)^*^	115 (97)
Cyclophosphamide (%)^*^	97 (82)
Intravenous (%)^*^	67 (73)
Oral (%)^*^	25 (27)
Cumulative dose (mg, median [IQR])^*^	4,000 [1,100; 6,112]
Rituximab (%)^*^	11 (9)
Other immunosuppressive agent (%)^*^	4 (3)

* *Presence of missing values*.

*IQR, interquartile range; ANCA, antineutrophil cytoplasm antibodies; CRP, C reactive protein; CT, computed tomography*.

The main symptoms at presentation were fatigue, fever, dyspnea, hemoptysis, and microscopic hematuria. One hundred and one (91%) had acute kidney injury at diagnostic with a median serum creatinine level of 7.2 mg/dl. Microscopic hematuria was found in 98% of patients, leukocyturia in 93%, and median proteinuria was 1.76 g/l. Fifty-four patients had alveolar hemorrhage confirmed by chest CT in 40 patients and bronchoalveolar lavage in 23 patients. Forty-eight individuals (40%) had combined kidney and lung involvement whereas 64 (54%) and 7 (6%) had isolated renal or pulmonary involvement, respectively.

Diagnosis of anti-GBM disease was assessed by the presence of anti-GBM antibodies (*n* = 103, 93%) and/or by renal histology revealing linear glomerular basement IgG deposits (*n* = 91, 99%) when tested.

One third of patients was admitted in an intensive care unit, 8 of them required mechanical ventilation, and 3 needed a vasopressor support. Initial renal replacement therapy was required in 91 patients (78%). Ninety-seven patients (82%) received plasma exchanges. The non-use of plasma exchange was most often decided in cases of advanced renal damage with scarring. Among the 115 patients who received tapering doses of oral prednisone, 81 also received 1 to 3 intravenous pulses of methylprednisolone (70%). A total of 97 (82%) individuals received CYC, intravenously in two-thirds of cases. Rituximab therapy was initiated within 3 months following the diagnosis in 11 (9%) patients. Four patients received other immunosuppressive agents (azathioprine, *n* = 3, mycophenolate mofetil, *n* = 1).

### Comparison of Anti-GBM Patients According to ANCA Status

Of the 107 patients tested, 30 were positive for ANCA (ANCA+, 28%), with anti-MPO specificity in the majority of cases (27/30). ANCA positive (ANCA+) patients were significantly older (median age 70 vs. 47 years-old, *p* < 0.0001), were less likely smokers (26 vs. 54%, *p* = 0.03), and cannabis users (0 vs. 7%) compare to ANCA negative (ANCA-) patients (Table 2). All of ANCA+ patients had acute renal failure at diagnosis. Conversely, only 4 (14%) of ANCA+ presented hemoptysis compared to 24 (32%) of ANCA- patients.

**Table 2:** Comparison of anti-GBM patients according to ANCA status.

	**ANCA – (*n* = 77)**	**ANCA + (*n* = 30)**	***P*-value**
**Clinical features**			
Age (years, median [IQR])	47 [26; 62]	70 [57; 78]	**<** **0.0001**
Female (%)	35 (45)	17 (57)	0.39
Toxics			
Tobacco (%)^*^	40 (54)	6 (26)	**0.03**
Cannabis (%)^*^	5 (7)	0 (0)	0.33
Other (%)	9 (12)	3 (10)	1
Comorbidities			
Hypertension (%)^*^	22 (29)	13 (46)	0.10
Diabetes (%)^*^	8 (10)	1 (3)	0.44
Dyslipidemia (%)^*^	7 (9)	7 (24)	0.055
**Renal involvement**			
Acute renal failure (%)^*^	64 (86)	30 (100)	0.059
Serum creatinine (mg/dl, median [IQR])^*^	7.3 [4.2; 10.4]	7.0 [3.8; 11.8]	0.74
Proteinuria (> 0.5 g/d, %)^*^	48 (89)	19 (100)	0.33
Microscopic hematuria (%)^*^	56 (98)	21 (100)	1
Leukocyturia (%)^*^	25 (89)	15 (100)	0.54
Serum albumin (g/l, median [IQR])^*^	26 [22; 31]	29 [25; 33]	0.40
Renal biopsy (%)	65 (84)	26 (87)	1
Extracapillary proliferation (%)^*^	48 (94)	14 (93)	1
Capsular rupture (%)^*^	23 (85)	9 (64)	0.23
Interstitial fibrosis (%)^*^	24 (60)	11 (69)	0.76
Hyaline thrombi (%)^*^	7 (15)	3 (15)	1
Immunofluorescence positivity (%)^*^	60 (100)	23 (96)	0.29
**Pulmonary involvement**			
Dyspnea (%)^*^	29 (39)	10 (34)	0.82
Cough (%)^*^	28 (37)	9 (31)	0.65
Overt hemoptysis (%)^*^	24 (32)	4 (14)	0.085
Pulmonary interstitial opacities on chest CT (*n*, %)^*^	27 (55)	12 (63)	0.60
Alveolar hemorrhage on bronchoalveolar lavage (*n*, %)^*^	15 (88)	7 (100)	1
PaO2 (mmHg, median [IQR])^*^	77 [60; 82]	81 [75; 93]	0.41
**Therapeutic regimens**			
Admission to intensive care (%)^*^	25 (32)	8 (28)	0.81
Mechanical ventilation (%)	5 (20)	3 (38)	0.37
Initial hemodialysis (%)^*^	57 (74)	24 (83)	0.45
Plasmapheresis (%)	64 (83)	25 (83)	1
Corticosteroid pulses (%)	56 (73)	19 (63)	0.36
Oral corticosteroids (%)	76 (99)	29 (97)	0.48
Cyclophosphamide (%)	63 (82)	25 (83)	1
Rituximab (%)	6 (8)	5 (17)	0.29
Other immunosuppressive agent (%)	3 (4)	1 (3)	1

* *Presence of missing values. Significant P-values are represented in bold. IQR, interquartile range; ANCA, antineutrophil cytoplasm antibodies; CRP, C reactive protein; CT, computer scan*.

Both groups had comparable rates of hospitalization in intensive care unit, with a higher rate of mechanical ventilation, vasopressor support, and hemodialysis in the ANCA+ group, although not statistically significant. Therapeutic regimens included plasma exchanges, corticosteroids, and cyclophosphamide in comparable rates. However, rituximab treatment was initiated in 17% of ANCA+ vs. only 8% of ANCA-, although this difference was not statistically significant.

### Overall Survival

**Figure 1:**
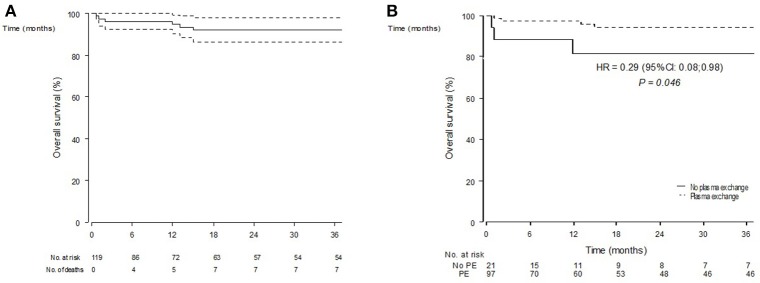
Overall survival estimates (Kaplan-Meier estimator) in *n* = 119 included patients **(A)** and according to the initial use of plasma exchanges **(B)**.

The OS was 95% (95% CI: 90–99) at 1 year and 92% (95% CI: 86–98) at 3 and 5 years (Figure 1A). Median survival was not reached during a median follow-up of 24 months (6–54). Eleven patients died during this follow-up. Among those, the median time from presentation until death was 13 months (1.5–60), 4 patients died during the first 6 months, and 5 during the first year. The serum creatinine levels at presentation were >500 μmol/L for 9 of them. They all required hemodialysis within the first month and 5 had isolated renal involvement. Causes of death were infections in 2 patients, acute congestive heart failure in 1 patient, discontinuation of hemodialysis after cessation of treatment in 1 patient, neoplastic complications in 3 patients (1 pulmonary cancer at 104 months, 2 urothelial bladder cancers at 15 and 168 months, respectively) and bedridden condition in 1 patient. In the other cases, the cause of death was not specified.

OS prognostic factors are summarized in Table 3. In univariate analyses, older age at presentation [HR for 10 years: 4.10 (1.89–8.88) *p* = 0.0003], history of hypertension [HR 19.9 (2.52–157.2) *p* = 0.005], or dyslipidemia [HR 11.1 (2.72–45) *p* = 0.0008], and initial mechanical ventilation [HR 5.20 (1.02–26.4) *p* = 0.047] were associated to death. Conversely, plasma exchanges use was associated with a better survival [HR 0.29 (0.08–0.98) *p* = 0.046] (Figure 1B). Gender, alveolar hemorrhage, ANCA status or the use of an alternative immunosuppressor was not associated to death.

**Table 3:** Overall survival prognostic factors.

	**HR [95% CI]**	***P*-value**
**Clinical features**		
Age (HR for 10 years)	4.10 [1.89; 8.88]	**0.0003**
Male	1.02 [0.31; 3.34]	0.98
Toxics		
Tobacco	0.59 [0.17; 2.01]	0.40
Cannabis		0.50^*^
Other	1.37 [0.17; 11.0]	0.77
Comorbidities		
Hypertension	19.9 [2.52; 157.2]	**0.005**
Diabetes		0.51^*^
Dyslipidemia	11.1 [2.71; 45.0]	**0.0008**
Time between onset and diagnosis (HR for 1 month)	0.010 [0.000; 1.69]	0.078
**Biological features**		
ANCA positivity	3.01 [0.78; 11.7]	0.11
Hemoglobin level	0.87 [0.32; 2.36]	0.79
CRP (HR for 10 mg/l)	0.79 [0.46; 1.37]	0.41
**Renal involvement**		
Serum creatinine (HR for 1 mg/dl)	0.97 [0.86; 1.09]	0.57
Proteinuria (> 0.5 g/dl)		0.41^*^
Microscopic hematuria		0.67^*^
Serum albumin	1.22 [0.79; 1.89]	0.38
Renal biopsy		
Extracapillary proliferation	1.91 [0.23; 16.0]	0.55
Immunofluorescence positivity		0.88^*^
**Pulmonary involvement**		
Dyspnea	0.73 [0.19; 2.86]	0.66
Cough	0.52 [0.11; 2.45]	0.41
Alveolar hemorrhage	1.13 [0.34; 3.72]	0.84
**Therapeutic regimens**		
Admission to intensive care	1.67 [0.42; 6.56]	0.46
Mechanical ventilation	5.20 [1.02; 6.56]	**0.047**
Initial hemodialysis		0.092^*^
Plasmapheresis	0.29 [0.08; 0.98]	**0.046**
Corticosteroid pulses	0.73 [0.21; 2.50]	0.42
Cyclophosphamide	0.58 [0.15; 2.20]	0.42
Rituximab		0.33^*^
Other immunosuppressive agent		0.50^*^

* *P-values from Log Rank tests, due to limited number of events across groups defined by the candidate variables. Significant P-values (<0.05) are represented in bold. HR, hazard ratio; CI, confidence interval; ANCA, antineutrophil cytoplasm antibodies; CRP, C reactive protein*.

### Renal Survival

**Figure 2:**
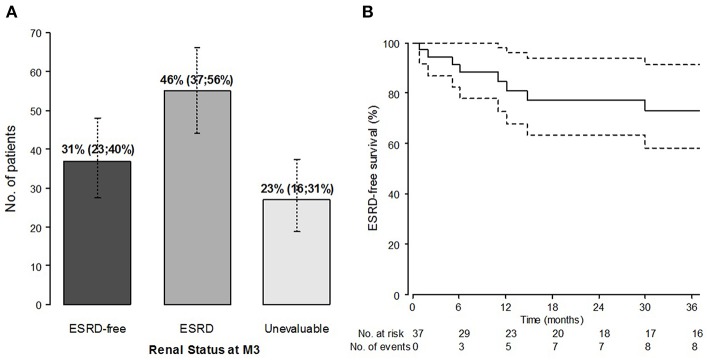
Renal outcome: prevalence of patients with ESRD at M3 (%, 95% confidence interval) **(A)** and ESRD-free survival (Kaplan-Meier estimates) from M3, in patients alive and without ESRD at M3 (*n* = 37) **(B)**.

We examined the baseline characteristics according to renal status, as diagnosed after 3 months of follow-up: ESRD+ (*n* = 55, 46%), ESRD- patients (*n* = 37, 31%), or not evaluable [follow-up shorter than 3 months, lost-to-follow-up (LFUP), *n* = 27, 23%; Figure 2A]. The ESRD+ and ESRD- group data are summarized in Table 4. The complete table including the LFUP group data is available in Supplementary Material. ESRD+ patients were older than ESRD- patients (57 vs. 37 years, *p* = 0.003). The biological parameters were similar including the positivity of the ANCAs. Serum creatinine level at presentation was significantly higher in ESRD+ patients than in ESRD- [9.1 (6.3; 14.3) vs. 4.0 mg/dl (1.4; 5.9), *p* < 0.0001]. The histological parameters seemed also associated with short-term renal impairment, although not statistically significant at the pre-defined threshold, with greater observed proportions of extracapillary proliferation (73 vs. 60%), capsular rupture (89 vs. 55%), interstitial fibrosis (69 vs. 56%), and hyaline thrombi (19 vs. 10%). Conversely, the initial pulmonary involvement seemed more frequent in ESRD- patients with more cough (58 vs. 23%, *p* = 0.004) and alveolar hemorrhage (71 vs. 36%) than in ESRD+. Concerning initial treatments, ESRD+ had required more frequently renal replacement therapy at the onset (96 vs. 44% in ESRD-, *p* < 0.0001), and tended to receive less CYC (76 vs. 94%, *p* = 0.060) than ESRD-.

**Table 4:** Comparison of anti-GBM patients according to ESRD status at M3 (ESRD status was categorized in 3 groups: ESRD–, ESRD+, Not evaluable [FUP < 3 months]).

**Variables**	**ESRD– (*n* = 37)**	**ESRD+ (*n* = 55)**	***P*-value^†^**
**Clinical features**			
Age (years)	37 [25; 56]	57 [38; 74]	**0.003**
Female (%)	15 (41)	31 (56)	0.35
Toxics			
Tobacco (%)^*^	20 (57)	22 (43)	0.22
Cannabis (%)^*^	3 (9)	1 (2)	0.34
Other (%)	5 (14)	2 (4)	0.057
Comorbidities			
Hypertension (%)^*^	11 (31)	19 (35)	0.88
Diabetes (%)^*^	3 (8)	2 (4)	0.15
Dyslipidemia (%)^*^	4 (11)	6 (11)	0.87
Time between onset and diagnosis (months, median [IQR])^*^	0.5 [0.1; 1.0]	0.3 [0.1; 0.8]	0.32
Biological features^*^			
ANCA positivity (%)	8 (24)	14 (29)	0.70
Hemoglobin level (g/dl)^*^	9 [8; 10]	9 [8; 10]	0.70
CRP (mg/L)^*^	84 [28; 142]	128 [86; 239]	0.044
**Renal involvement**			
Serum creatinine (mg/dl)^*^	4.0 [1.4; 5.9]	9.1 [6.4; 14.3]	**<** **0.0001**
Proteinuria (> 0.5 g/dl, %)^*^	25 (86)	27 (96)	0.43
Microscopic hematuria (%)^*^	31 (97)	28 (100)	0.74
Leukocyturia (%)^*^	16 (89)	12 (100)	0.77
Serum albumin (g/l)^*^	30 [22; 33]	26 [23; 31]	0.25
Renal biopsy (%)^*^	30 (83)	50 (93)	
Extracapillary proliferation (%)^*^	18 (60)	37 (74)	0.41
Capsular rupture (%)^*^	6 (55)	16 (89)	0.12
Interstitial fibrosis (%)^*^	6 (56)	20 (69)	0.64
Hyaline thrombi (%)^*^	2 (10)	7 (19)	0.69
Immunofluorescence positivity (%)^*^	29 (97)	46 (100)	0.50
**Pulmonary involvement**			
Dyspnea (%)^*^	16 (50)	16 (30)	0.20
Cough (%)^*^	19 (58)	12 (23)	**0.004**
Alveolar hemorrhage (%)^*^	25 (71)	19 (36)	**0.002**
**Therapeutic regimens**			
Admission to intensive care (%)^*^	8 (24)	15 (27)	0.11
Mechanical ventilation (%)	5 (62)	2 (13)	**0.015**
Initial hemodialysis (%)^*^	15 (44)	53 (96)	**<** **0.0001**
Plasmapheresis (%)^*^	32 (89)	44 (80)	0.45
Corticosteroid pulses (%)^*^	25 (69)	36 (68)	0.84
Cyclophosphamide (%)^*^	34 (94)	42 (76)	0.06
Rituximab (%)^*^	6 (17)	3 (5)	0.23
Other immunosuppressive agent (%)^*^	2 (6)	2 (4)	0.69

* *Presence of missing values*.

† *P-values for Fisher's exact tests or Kruskal-Wallis tests comparing discrete and continuous variables, respectively, across ESRD+, ESRD–, and LFUP (lost-to-follow-up before 3 months) groups. Significant P-values (<0.05) are represented in bold. ANCA, antineutrophil cytoplasm antibodies; CRP, C reactive protein*.

The majority of patients presented with severe renal failure at diagnosis. However, of the 50 patients with a serum creatinine level of <6.8 mg/dl (i.e., 600 μmol/L) at diagnosis, 26 were nevertheless dialyzed immediately because of a rapid degradation of their renal function. The description of the cohort according to the creatinine level (< or ≥ 6.8 mg/dl) and the Kaplan-Meier curves for overall survival by group are available in Supplementary Material.

Ninety-one (78%) patients required dialysis at presentation. Of these, 53 progressed to chronic end stage renal failure (ESDR+), 15 have recovered renal function (ESRD-), and 23 have been lost to follow-up (LFUP) at M3. ESRD- patients at M3 had a lower serum creatinine at presentation [6.1 mg/dl (6.1;12.1) vs. 9.8 (6.5;14.6), *p* = 0.006], were less likely to have hypertension at diagnosis (29 vs. 46%, *p* = 0.024), had more often pulmonary involvement (hemoptysis 47 vs. 18%, *p* = 0.019; alveolar hemorrhage 73 vs. 37%, *p* = 0.022) and have more often required the use of mechanical ventilation (100 vs. 13%, *p* = 0.001) than ESRD+ patients (Supplementary Material).

ESRD-free survival in patients without ESRD alive at M3 (*n* = 37) is represented in Figure 2B. Starting from M3, the median follow-up was 44 months (9–81). During the follow-up, 10 of the 37 M3-ESRD- patients eventually developed ESRD, following the adverse course of renal function or relapse of the disease; two of them died. In the M3-ESRD-population, ESRD-free survival prognostic factors are presented in Table 5. The main predictors of poor renal outcome were: the presence of hyaline thrombi on renal biopsy [HR 17 (95% confidence interval (CI) 1.06; 271.6) *p* = 0.045]; and cannabis use [HR 7.64 (1.80; 32.5) *p* = 0.006].

**Table 5:** ESRD-free survival prognostic factors, in ESRD-free patients alive at M3 (*n* = 37).

	**HR [95% CI]**	***P-*value**
**Clinical features**		
Age (HR for 10 years)	1.02 [0.67; 15.7]	0.14
Male	3.24 [0.67; 1.45]	0.91
Toxics		
Tobacco	1.00 [0.27; 3.74]	1
Cannabis	7.64 [1.80; 32.5]	**0.006**
Other	0.9 [0.11; 7.32]	0.92
Comorbidities		
Hypertension	0.66 [0.14; 3.17]	0.60
Diabetes		0.29^*^
Dyslipidemia	1.20 [0.15; 9.59]	0.87
Time between onset and diagnosis (HR for 1 month)	0.76 [0.42; 1.40]	0.38
**Biological features**		
ANCA positivity	1.15 [0.23; 5.74]	0.86
Hemoglobin level	1.34 [0.68; 2.64]	0.40
CRP (HR for 10 mg/L)	1.01 [0.83; 1.23]	0.92
**Renal involvement**		
Serum creatinine (HR for 1 mg/dl)	1.01 [0.80; 1.27]	0.95
Proteinuria (> 0.5 g/dl)		0.35^*^
Microscopic hematuria		0.49^*^
Leukocyturia		0.35^*^
Serum albumin	0.91 [0.77; 1.09]	0.31
Renal biopsy		
Extracapillary proliferation	0.76 [0.18; 3.22]	0.71
Capsular rupture	0.82 [0.05; 13.2]	0.89
Interstitial fibrosis	3.11 [0.34; 28.7]	0.32
Hyaline thrombi	17 [1.06; 271.6]	**0.045**
Immunofluorescence positivity		0.73^*^
**Pulmonary involvement**		
Dyspnea	1.26 [0.31; 5.08]	0.75
Cough	2.99 [0.60; 14.9]	0.18
Alveolar hemorrhage	3.81 [0.47; 30.5]	0.21
**Therapeutic regimens**		
Admission to intensive care	1.43 [0.29; 7.16]	0.66
Mechanical ventilation	2.73 [0.55; 13.6]	0.22
Initial hemodialysis	1.72 [0.46; 6.44]	0.42
Plasmapheresis	0.30 [0.06; 1.48]	0.14
Corticosteroid pulses	1.62 [0.34; 7.84]	0.55
Cyclophosphamide		0.45^*^
Rituximab		0.18^*^
Other immunosuppressive agent	1.57 [0.20; 12.6]	0.67

* *P-values from Log Rank tests, due to limited number of events across groups defined by the candidate variables. Significant P-values (<0.05) are represented in bold. HR, hazard ratio; CI, confidence interval; ANCA, antineutrophil cytoplasm antibodies; CRP, C reactive protein*.

At the end of the follow-up, among all patients who reached ESRD (*n* = 62, 67%), 29 patients were still in hemodialysis and 33 had received kidney transplant. Five patients (4%) had a relapse during the follow-up with a median of 12 months following diagnosis. Among them, two were renal relapses, one pulmonary relapse, and one affecting both organs. All pulmonary relapses involved patients with isolated lung involvement. Relapsing patients received therapeutic regimen including 4/5 (80%) plasma exchanges, 5/5 (100%) corticosteroids, 4/5 (80%) CYC, and none received rituximab. No relapse was observed after transplantation.

## Discussion

Anti-GBM disease is a rare disease with an estimated incidence between 0.5 and 1.6 case per million per year (23) but it represents 1 to 5% of all types of glomerulonephritis and ~20% of rapidly progressive glomerulonephritis (24–26). The severity of disease imposes an early diagnosis to initiate rapidly plasmapheresis and immunosuppressive treatments. There are still unmet needs to identify prognostic factors prior to complications to target patients needing more aggressive therapy.

In this large French nationwide multicenter cohort, we first analyzed anti-GBM patients according to ANCA status. ANCA positivity was found in 28% of patients. Double-positive patients were older, less frequently smokers, and had less pulmonary involvement. Consistently with previous series (7, 10, 27), we reported a high frequency of ANCA positive anti-GBM disease patients. Olson et al. (28) suggest that ANCA-induced glomerular inflammation may be a trigger for the development of an anti-GBM response, perhaps by modifying or exposing usually sequestered disease epitopes in GBM, since it has been shown that ANCA may be detected before the onset of anti-GBM disease. Our ANCA positive patients experienced severe renal involvement since all of them presented acute renal failure at onset compared to 86% of their ANCA negative counterpart. In contrast, lung involvement was less frequent. In a large European cohort, McAdoo et al showed that double-positive patients had severe kidney and lung disease at presentation, requiring aggressive immunosuppressive therapy, and plasma exchange (10). During long-term follow-up, they relapsed at a frequency comparable to a parallel cohort of patients with ANCA-associated vasculitis (AAV), suggesting they warrant more careful long-term follow-up and maintenance immunosuppression, unlike patients with single-positive anti-GBM disease.

The presence of hyaline thrombi on renal biopsy and cannabis use were significantly associated with ESRD in patients without initial ESRD. In our study, ESRD at 3 months was observed in 46% of cases. ESRD positive patients were older, more frequently men, and had higher serum creatinine level at presentation than those without ESRD. These results are consistent with those of previous studies showing that the occurrence of oliguria or anuria, elevated serum creatinine at presentation and the percentage of crescents were shown to be risk factors for ESRD (19, 29).

This large cohort allowed us to identify four prognostic factors of overall survival. We identified age at onset, existence of cardiovascular risk factors, aggressiveness of initial management with mechanical ventilation, and the absence of plasmapheresis as significantly associated with death in anti-GBM patients. Mortality in anti-GBM used to be extremely high, up to 95% in older series (30) and was mainly related to pulmonary hemorrhage, or to end-stage renal failure. New protocols including plasmapheresis, glucocorticoids, and cyclophosphamide (CYC) had dramatically improved patient's outcomes. In our study, the 1 and 5-year survival reached 95 and 92%, respectively. This rate was higher than OS observed in recent other series. Proskey et al. reported 88% survival rate in an English study over 20 years (14). Huart et al reported 86.9% 1-year survival rate (20). This improvement of survival rate could be explained by the relatively low rate of infectious complications (23%), and severe infections accounted only for 2 out of 11 deaths. In contrast, 3 deaths were attributable to cancers (at 15, 108, and 162 months after presentation, respectively), and 2 others occurred after renal transplantation. This underlines the need to take into account the toxicity of immunosuppressive treatments (mainly CYC) used in the acute phase of the disease, and anti-rejection treatments after transplantation. In this respect, induction therapy with rituximab, may reduce the risk of developing secondary cancer.

Surprisingly, pulmonary involvement was not a factor of poor prognosis. Our study confirms the results of other series on the importance of plasma exchanges positively associated with overall survival. Huart et al. showed that a cut-off of 8 plasmapheresis sessions was associated with positive and negative predictive survival rates of 95 and 47%, respectively (20). Given the physiopathological importance of the clearance of autoantibodies in the disease, the number of plasma exchanges could be monitored according to the course of circulating anti-GBM levels. On 111 patients tested, 8 (7%) were antibody-negative anti-GBM disease. Seven of them had acute renal failure and half had alveolar hemorrhage at presentation. These results differ somewhat from those of a recent study reporting 19 cases of negative antibody patients with better renal function at biopsy and less lung involvement than in classic anti-GBM patients (31).

We acknowledge some limitations in our study. Our analysis was performed in a retrospective manner. We were unable to collect complete longitudinal data on patients who were seen only on an intermittent consultation basis. A few initial patients (27/119, 23%) were lost-to follow-up soon after diagnosis, before M3, most often due to a change of medical center for dialysis or pre-transplant assessment. However, to prevent selection biases, these patients, categorized at unevaluable at M3 for renal function, were included in the descriptive analysis and evaluating prognostic factors of ESRD status at M3 (Supplementary Material). Furthermore, given that ESRD diagnosis requires a 3-month follow-up time window by definition, we used a landmark approach to examine prognostic factors of ERD-free survival from M3. The sample was therefore restricted to ESRD-free patients alive at M3 (*n* = 37) and limited in size; nonetheless, the landmark approach is an adequate approach to prevent immortal time bias (22). The quantification of diuresis and the evolution of urinary sediment would have let us evaluate anuria and proteinuria as potential pejorative prognostic factors of renal evolution. Our study also comes up against the lack of proofreading of anatomopathological features of renal biopsies. The presence of hyaline thrombi remains a non-specific element, especially in acute glomerulonephritis. We could not specify either their location or their number. Similarly, the presence of acute tubular necrosis lesions and/or vasculitis has not been reported. In a recent study classifying 123 anti-GBM kidney biopsy samples according to ANCA-associated GN, histopathological class, and kidney survival were associated. Low percentage of normal glomeruli and large extent of interstitial infiltrate were associated with poor renal survival (32). Anti-GBM antibodies levels could only very rarely be collected during follow-up. Thus, their rate after plasma exchange was available for only 47% of patients, limiting the interpretation of the impact of treatments on the clearance of autoantibodies. Although we provide univariate analyses of EFS and of OS, due to the limited number of events, we were unable to perform robust multivariate analyzes for these outcomes (33). Prospective enrollment and data collection from the time of diagnosis would have been ideal but are difficult to achieve with such rare diseases.

In conclusion, this French nationwide study shows that older age at diagnosis, female gender, a high serum creatinine level at presentation, and extracapillary proliferation predicted renal survival in patients with anti-GBM disease. We identified age at onset, existence of cardiovascular risk factors, aggressiveness of initial management with mechanical ventilation and the absence of plasmapheresis as significantly associated with death in anti-GBM patients.

## Author Contributions

CM, JC, NJ-C, and DS contributed conception and design of the study. CM, JC, and AP organized the database. LB performed the statistical analysis. CM wrote the first draft of the manuscript. All authors contributed to manuscript revision, read, and approved the submitted version.

### Conflict of Interest Statement

The authors declare that the research was conducted in the absence of any commercial or financial relationships that could be construed as a potential conflict of interest.
