# Supplementary material for: Prognostic Factors in Anti-glomerular Basement Membrane Disease: A Multicenter Study of 119 Patients
Source: Front Immunol. 2019 Jul 18;10:1665. doi: 10.3389/fimmu.2019.01665 (PMC6662558; doi:10.3389/fimmu.2019.01665)
Supplement: Supplementary file 1 [file Data_Sheet_1.docx]

**Supplemental Material to**

**Prognostic factors in anti-glomerular basement membrane disease: a multicenter study of 119 patients**

Cindy Marques MD1*, Julien Carvelli MD2*, Lucie Biard PhD3, Stanislas Faguer MD PhD4, François Provôt, MD5, Marie Matignon MD6, Jean-Jacques Boffa MD PhD7, Emmanuelle Plaisier MD PhD7, Alexandre Hertig MD PhD7, Maxime Touzot MD8, Olivier Moranne MD PhD9, Xavier Belenfant MD10, Djillali Annane MD PhD11, Thomas Quemeneur MD12, Jacques Cadranel MD PhD13, Hassan Izzedine MD14, Nicolas Bréchot MD15, Patrice Cacoub MD PhD1, Alexandre Piedrafita MD4, Noémie Jourde-Chiche MD2†, David Saadoun MD PhD1†

**Table S1. Comparison of anti-GBM patients according to ESRD status at M3** (ESRD status was categorized in 3 groups: ESRD-, ESRD+, Not evaluable [FUP < 3 months]).

|  | ESRD-  (n = 37) | ESRD+  (n = 55) | Unavailable status  (n=27, 23%) | P-value |
| --- | --- | --- | --- | --- |
| **Clinical features** |  |  |  |  |
| Age (years) | 37 [25 ; 56] | 57 [38 ; 74] | 65 [40;76] | **0.003** |
| Female (%) | 15 (41) | 31 (56) | 13 (48) | 0.35 |
| Toxics |  |  |  |  |
| Tobacco (%)* | 20 (57) | 22 (43) | 8 (35) | 0.22 |
| Cannabis (%)* | 3 (9) | 1 (2) | 2 (9) | 0.34 |
| Other (%) | 5 (14) | 2 (4) | 5 (19) | 0.057 |
| Comorbidities |  |  |  |  |
| Hypertension (%)* | 11 (31) | 19 (35) | 10 (37) | 0.88 |
| Diabetes (%)* | 3 (8) | 2 (4) | 4 (15) | 0.15 |
| Dyslipidemia (%)* | 4 (11) | 6 (11) | 4 (15) | 0.87 |
| Time between onset and diagnosis (months, median [IQR])* | 0.5 [0.1 ; 1.0] | 0.3 [0.1 ; 0.8] | 0.6 [0.2;1.2] | 0.32 |
| **Biological features*** |  |  |  |  |
| ANCA positivity (%) | 8 (24) | 14 (29) | 8 (32) | 0.70 |
| Hemoglobin level (g/dl)* | 9 [8 ; 10] | 9 [8 ; 10] | 9 [8 ; 10] | 0.70 |
| CRP (mg/L)* | 84 [28 ; 142] | 128 [86 ; 239] | 71 [30;138] | 0.044 |
| **Renal involvement** |  |  |  |  |
| Serum creatinine (mg/dl)* | 4.0 [1.4 ; 5.9] | 9.1 [6.4 ; 14.3] | 8.5 [5.3;11.9] | **<0.0001** |
| Proteinuria (> 0,5g/dl, %)* | 25 (86) | 27 (96) | 20 (91) | 0.43 |
| Microscopic hematuria (%)* | 31 (97) | 28 (100) | 22 (96) | 0.74 |
| Leukocyturia (%)* | 16 (89) | 12 (100) | 14 (93) | 0.77 |
| Serum albumin (g/l)* | 30 [22 ; 33] | 26 [23 ; 31] | 26 [22;29] | 0.25 |
| Renal biopsy (%)* | 30 (83) | 50 (93) | 21 (78) |  |
| Extracapillary proliferation (%)* | 18 (60) | 37 (74) | 14 (67) | 0.41 |
| Capsular rupture (%)* | 6 (55) | 16 (89) | 10 (77) | 0.12 |
| Interstitial fibrosis (%)* | 6 (56) | 20 (69) | 8 (67) | 0.64 |
| Hyaline thrombi (%)* | 2 (10) | 7 (19) | 2 (15) | 0.69 |
| Immunofluorescence positivity (%)* | 29 (97) | 46 (100) | 16 (100) | 0.50 |
| **Pulmonary involvement** |  |  |  |  |
| Dyspnea (%)* | 16 (50) | 16 (30) | 10 (38) | 0.20 |
| Cough (%)* | 19 (58) | 12 (23) | 8 (31) | **0.004** |
| Alveolar hemorrhage (%)* | 25 (71) | 19 (36) | 10 (37) | **0.002** |
| **Therapeutic regimens** |  |  |  |  |
| Admission to intensive care (%)* | 8 (24) | 15 (27) | 13 (48) | 0.11 |
| Mechanical ventilation (%) | 5 (62) | 2 (13) | 1 (8) | **0.015** |
| Initial hemodialysis (%)* | 15 (44) | 53 (96) | 23 (85) | **<0.0001** |
| Plasmapheresis (%)* | 32 (89) | 44 (80) | 21 (78) | 0.45 |
| Corticosteroid pulses (%)* | 25 (69) | 36 (68) | 20 (74) | 0.84 |
| Cyclophosphamide (%)* | 34 (94) | 42 (76) | 21 (78) | 0.06 |
| Rituximab (%)* | 6 (17) | 3 (5) | 2 (7) | 0.23 |
| Other immunosuppressive agent (%)* | 2 (6) | 2 (4) | 0 (0) | 0.69 |

* Presence of missing values

Significant P values (<0.05) are represented in bold.

*ANCA*, antineutrophil cytoplasm antibodies; *CRP*, C reactive protein

**Table S2. Description of patients according to baseline serum creatinine level (<6.8 mg/dl vs. ≥ 6.8 mg/dl).** *(n=111, 8 patients with missing data for baseline creatinine* *level).*

|  | **Creatinine < 6.8 mg/dl** | **Creatinine ≥ 6.8 mg/dl** |
| --- | --- | --- |
| **Patients, n.** | **50** | **61** |
| **Clinical features** |  |  |
| Age (years, median [IQR]) | 53 [31 ; 72] | 57 [36 ; 74] |
| Female (%) | 28 (56) | 29 (48) |
| Ethnic group* |  |  |
| Caucasian (%) | 38 (84) | 51 (85) |
| Other (%) | 7 (16) | 9 (15) |
| Toxics |  |  |
| Tobacco (%)* | 21 (51) | 25 (42) |
| Cannabis (%)* | 4 (10) | 1 (2) |
| Other (%) | 6 (12) | 6 (10) |
| Comorbidities |  |  |
| Hypertension (%)* | 17 (35) | 21 (34) |
| Diabetes (%)* | 5 (10) | 3 (5) |
| Dyslipidemia (%)* | 10 (20) | 4 (7) |
| Time between onset and diagnosis (months, median [IQR]) | 0.7 [0.1 ;1.7] | 0.2 [0.1 ; 0.7] |
| Symptom leading to the medical consultation* |  |  |
| Fatigue (%) | 13 (27) | 25 (41) |
| Fever (%) | 4 (8) | 4 (7) |
| Dyspnea (%) | 5 (10) | 6 (10) |
| Cough (%) | 6 (12) | 1 (2) |
| Hemoptysis (%) | 9 (18) | 6 (10) |
| Microscopic hematuria (%) | 3 (6) | 6 (10) |
| Biological anomaly (%) | 9 (18) | 13 (21) |
| **Biological features** |  |  |
| ANCA positivity (%)* | 12 (25) | 18 (32) |
| Hemoglobin level (g/dl, median [IQR])* | 9 (8;10) | 9 (8;10) |
| CRP (mg/L, median [IQR])* | 93 (43;165) | 83 (30;158) |
| **Renal involvement** |  |  |
| Acute renal failure (%)* | 40 (80) | 61 (100) |
| Serum creatinine (mg/dl, median [IQR])* | 3.8 [1.5;5.7] | 11.0 [8.5;14.0] |
| Proteinuria (> 0,5g/dl, %)* | 36 (86) | 36 (97) |
| Microscopic hematuria (%)* | 42 (95) | 37 (100) |
| Leukocyturia (%)* | 24 (89) | 18 (100) |
| Serum albumin (g/l, median [IQR])* | 29 [25;33] | 25 [22;29] |
| Renal biopsy (%)* | 40 (80) | 54 (90) |
| Extracapillary proliferation (%)* | 22 (96) | 43 (93) |
| Capsular rupture (%)* | 14 (70) | 17 (81) |
| Interstitial fibrosis (%)* | 12 (55) | 24 (71) |
| Hyaline thrombi (%)* | 2 (7) | 9 (23) |
| Immunofluorescence positivity (%)* | 37 (97) | 48 (100) |
| **Pulmonary involvement** |  |  |
| Dyspnea (%)* | 21 (44) | 21 (35) |
| Cough (%)* | 23 (47) | 16 (27) |
| Overt hemoptysis (%)* | 18 (37) | 11 (18) |
| Pulmonary interstitial opacities on chest CT (n, %)* | 22 (56) | 18 (58) |
| Alveolar hemorrhage on bronchoalveolar lavage (n, %)* | 14 (100) | 9 (90) |
| PaO2 (mmHg, median [IQR])* | 78 [60;87] | 76 [61;81] |
| **Therapeutic regimens** |  |  |
| Admission to intensive care (%)* | 15 (31) | 20 (33) |
| Mechanical ventilation (%) | 5 (33) | 3 (15) |
| Initial hemodialysis (%)* | 26 (53) | 59 (97) |
| Plasmapheresis (%)* | 42 (84) | 50 (82) |
| Corticosteroid pulses (%)* | 32 (64) | 45 (75) |
| Oral corticosteroids (%)* | 50 (100) | 59 (97) |
| Cyclophosphamide (%)* | 44 (88) | 49 (80) |
| Rituximab (%)* | 8 (16) | 3 (5) |
| Other immunosuppressive agent (%)* | 3 (6) | 1 (2) |
| **ESRD status at M3** |  |  |
| ESRD- | 27 (54%) | 6 (10%) |
| ESRD+ | 14 (28%) | 38 (62%) |
| Lost-to-follow up at 3 months (%) | 9 (18) | 17 (28) |

* Presence of missing values

**Figure S1. Kaplan Meier-estimated Overall Survival by baseline serum creatinine level (<6.8 mg/dl vs. ≥ 6.8 mg/dl).**

Overall survival (%)

0

6

12

18

24

30

36

0

20

40

60

80

100

Time (months)

No. at risk

Creat. < 6.8 mg/dl

Creat. >= 6.8 mg/dl

50

61

39

40

30

35

27

30

23

28

22

26

22

26

Creatinine < 6.8 mg/dl

Creatinine >= 6.8 mg/dl

*Log Rank P = 0.44*

At 36 months, OS was 94% (95%confidence interval: 85;100%) in patients with initial creatinine < 6.8 mg/dl and 89% (95%CI: 81;99%) in patients with initial creatinine ≥ 6.8 mg/dl.

**Table S3. Description of patients according to baseline need for dialysis (at presentation).** *(n=116, 3 patients with missing data for need for dialysis).*

| **Variables** | **No dialysis at presentation** | **Need for dialysis at presentation** |
| --- | --- | --- |
| **Patients, n. (%)** | **25** | **91** |
| **Clinical features** |  |  |
| Age (years, median [IQR]) | 36 [26 ; 52] | 58 [36 ; 75] |
| Female (%) | 10 (40) | 48 (53) |
| Ethnic group* |  |  |
| Caucasian (%) | 19 (83) | 74 (85) |
| Other (%) | 4 (17) | 13 (14) |
| Toxics |  |  |
| Tobacco (%)* | 13 (54) | 36 (43) |
| Cannabis (%)* | 4 (17) | 2 (2) |
| Other (%) | 6 (12) | 6 (10) |
| Comorbidities |  |  |
| Hypertension (%)* | 4 (16) | 35 (39) |
| Diabetes (%)* | 1 (4) | 8 (9) |
| Dyslipidemia (%)* | 2 (8) | 12 (13) |
| Time between onset and diagnosis (months, median [IQR]) | 0.7 [0.2 ; 2.6] | 0.3 [0.1 ; 0.9] |
| Symptom leading to the medical consultation* |  |  |
| Fatigue (%) | 5 (20) | 33 (37) |
| Fever (%) | 1 (4) | 9 (10) |
| Dyspnea (%) | 2 (8) | 9 (10) |
| Cough (%) | 4 (16) | 3 (3) |
| Hemoptysis (%) | 7 (28) | 8 (9) |
| Microscopic hematuria (%) | 1 (4) | 8 (9) |
| Biological anomaly (%) | 5 (20) | 20 (22) |
| **Biological features** |  |  |
| ANCA positivity (%)* | 5 (20) | 24 (30) |
| Hemoglobin level (g/dl, median [IQR])* | 9 [7;10] | 9 [8;10] |
| CRP (mg/L, median [IQR])* | 72 [28;127] | 96 [43;165] |
| **Renal involvement** |  |  |
| Acute renal failure (%)* | 15 (60) | 85 (100) |
| Serum creatinine (mg/dl, median [IQR])* | 2.1 [1.0;3.9] | 8.6 [6.1;12.1] |
| Proteinuria (> 0,5g/dl, %)* | 19 (83) | 52 (95) |
| Microscopic hematuria (%)* | 22 (96) | 58 (98) |
| Leukocyturia (%)* | 13 (81) | 28 (100) |
| Serum albumin (g/l, median [IQR])* | 31 [26;33] | 26 [22;29] |
| Renal biopsy (%)* | 19 (76) | 81 (90) |
| Extracapillary proliferation (%)* | 11 (92) | 58 (95) |
| Capsular rupture (%)* | 6 (67) | 26 (79) |
| Interstitial fibrosis (%)* | 3 (27) | 35 (73) |
| Hyaline thrombi (%)* | 1 (7) | 10 (18) |
| Immunofluorescence positivity (%)* | 18 (95) | 72 (100) |
| **Pulmonary involvement** |  |  |
| Dyspnea (%)* | 11 (46) | 31 (36) |
| Cough (%)* | 12 (48) | 27 (31) |
| Overt hemoptysis (%)* | 13 (52) | 18 (20) |
| Pulmonary interstitial opacities on chest CT (n, %)* | 14 (64) | 25 (53) |
| Alveolar hemorrhage on bronchoalveolar lavage (n, %)* | 6 (100) | 16 (89) |
| PaO2 (mmHg, median [IQR])* | 79 [69;89] | 75 [60;81] |
| **Therapeutic regimens** |  |  |
| Admission to intensive care (%)* | 4 (16) | 32 (36) |
| Mechanical ventilation (%) | 1 (25) | 7 (22) |
| Plasmapheresis (%)* | 21 (84) | 74 (81) |
| Corticosteroid pulses (%)* | 16 (64) | 64 (72) |
| Oral corticosteroids (%)* | 25 (100) | 88 (97) |
| Cyclophosphamide (%)* | 22 (88) | 73 (80) |
| Rituximab (%)* | 3 (12) | 8 (9) |
| Other immunosuppressive agent (%)* | 2 (8) | 2 (2) |

* Presence of missing values

**Figure S2. Kaplan Meier-estimated Overall Survival by baseline need for dialysis, at presentation (n=116)**

At 36 months, OS was 100% (no deqths) in patients without hemodialysis and 89% (95%CI: 82;97%) in patients who required hemodialysis at presentation.

**Table S4. Comparison of anti-GBM patients needing hemodialysis at diagnosis according to ESRD status at M3** (ESRD status was categorized in 3 groups: ESRD-, ESRD+, Not evaluable [FUP < 3 months]).

|  | Total  (n = 91) | ESRD-  (n = 15) | ESRD+  (n = 53) | Unavailable status  (n = 23) | P-value |
| --- | --- | --- | --- | --- | --- |
| **Clinical features** |  |  |  |  |  |
| Age (years) | 58 (36;75) | 50 (28;64) | 57 (36;73) | 71 (50;76) | 0.10 |
| Female (%) | 48 (53) | 6 (40) | 31 (58) | 11 (48) | 0.41 |
| Toxics |  |  |  |  |  |
| Tobacco (%)* | 36 (43) | 7 (50) | 22 (45) | 7 (35) | 0.64 |
| Cannabis (%)* | 2 (2) | 0 (0) | 1 (2) | 1 (5) | 0.66 |
| Comorbidities |  |  |  |  |  |
| Hypertension (%)* | 35 (39) | 6 (40) | 19 (37) | 10 (43) | 0.87 |
| Diabetes (%) | 8 (9) | 2 (13) | 2 (4) | 4 (17) | 0.087 |
| Dyslipidemia (%) | 12 (13) | 2 (13) | 6 (11) | 4 (17) | 0.76 |
| **Biological features*** |  |  |  |  |  |
| ANCA positivity (%)* | 24 (30) | 4 (29) | 13 (28) | 7 (33) | 0.94 |
| Hemoglobin level (g/dl)* | 9 (8;10) | 9 (8;10) | 9 (8;10) | 9 (8;10) | 0.46 |
| CRP (mg/L)* | 96 (43;165) | 98 (42;148) | 118 (85;227) | 71 (30;138) | 0.15 |
| **Renal involvement** |  |  |  |  |  |
| Serum creatinine (mg/dl)* | 8.6 (6.1;12.1) | 6.1 (4.1;7.9) | 9.8 (6.5;14.6) | 10.3 (7.2;12.1) | **0.006** |
| Proteinuria (> 0,5g/dl, %)* | 52 (95) | 10 (91) | 25 (96) | 17 (94) | 0.78 |
| Microscopic hematuria (%)* | 58 (98) | 12 (100) | 28 (100) | 18 (95) | 0.53 |
| Serum albumin (g/l)* | 26 (22;29) | 28 (22;28) | 26 (23;30) | 26 (22;27) | 0.79 |
| Renal biopsy (%)* | 81 (90) | 14 (93) | 48 (92) | 19 (83) | 0.44 |
| Extracapillary proliferation (%) | 58 (72) | 10 (71) | 35 (73) | 13 (68) | 0.94 |
| Capsular rupture (%)* | 26 (79) | 2 (50) | 15 (88) | 9 (75) | 0.22 |
| Interstitial fibrosis (%)* | 35 (73) | 8 (80) | 20 (71) | 7 (70) | 0.91 |
| Hyaline thrombi (%)* | 10 (18) | 1 (11) | 7 (19) | 2 (17) | 1 |
| Hyperrtension at diagnosis (%)* | 42 (49) | 4 (29) | 23 (46) | 15 (71) | **0.024** |
| **Pulmonary involvement** |  |  |  |  |  |
| Dyspnea (%)* | 31 (36) | 7 (50) | 16 (31) | 8 (36) | 0.43 |
| Cough (%)* | 27 (31) | 8 (57) | 12 (24) | 7 (32) | 0.064 |
| Hemoptysis (%)* | 18 (20) | 7 (47) | 9 (18) | 2 (9) | **0.019** |
| Alveolar hemorrhage (%)* | 37 (42) | 11 (73) | 19 (37) | 7 (30) | **0.022** |
| **Therapeutic regimens** |  |  |  |  |  |
| Admission to intensive care (%)* | 32 (36) | 4 (29) | 15 (28) | 13 (57) | 0.060 |
| Mechanical ventilation (%) | 7 (22) | 4 (100) | 2 (13) | 1 (8) | **0.001** |
| Plasmapheresis (%) | 74 (81) | 15 (100) | 42 (79) | 17 (74) | 0.097 |
| Corticosteroid pulses (%)* | 64 (72) | 12 (80) | 35 (69) | 17 (74) | 0.77 |
| Cyclophosphamide (%) | 73 (80) | 15 (100) | 40 (75) | 18 (78) | 0.096 |
| Rituximab (%) | 8 (9) | 3 (20) | 3 (6) | 2 (9) | 0.22 |
| Other immunosuppressive agent (%) | 2 (2) | 1 (7) | 1 (2) | 0 (0) | 0.37 |

* Presence of missing values

Significant P values (<0.05) are represented in bold.

*ANCA*, antineutrophil cytoplasm antibodies; *CRP*, C reactive protein
